# Risk of disseminated tuberculosis and other infections after neonatal Bacillus Calmette–Guérin vaccination in infants with *in-utero* exposure to tumor necrosis factor-α inhibitors

**DOI:** 10.3389/fmed.2026.1812473

**Published:** 2026-06-08

**Authors:** Samar Al Emadi, Seham Alebbi, Nawal Hadwan, Omar Alsaed, Priyanka Moovara Cackamvalli, Nevin Abunahia, Eman Satti

**Affiliations:** 1 Department of Rheumatology, Hamad Medical Corporation, Doha, Qatar; 2 Department of Gastroenterology, Hamad Medical Corporation, Doha, Qatar

**Keywords:** autoimmune rheumatic diseases, BCG vaccination, infection, outcome, pregnancy, TNF-α inhibitors, tuberculosis

## Abstract

**Introduction:**

Infants born to mothers with autoimmune rheumatic diseases, including rheumatoid arthritis, spondyloarthropathy, and inflammatory bowel disease, may be exposed antenatally to tumor necrosis factor-alpha (TNF-α) inhibitors. Recent guidelines recommend deferring live attenuated vaccines, such as Bacillus Calmette–Guérin (BCG), until after 6–12 months of age in this population, owing to conflicting safety data. This study aimed to evaluate the safety of administering BCG vaccination at birth in infants exposed to TNF-α inhibitors in utero.

**Method:**

We retrospectively screened the electronic health records of women with autoimmune rheumatic diseases treated with TNF-α inhibitors during pregnancy. Only children with complete health records from infancy were included in the analysis. Data were obtained from the largest health system in Qatar. Information on obstetric history, type and duration of TNF-α inhibitor therapy during pregnancy, age at BCG vaccine administration, adverse reactions to the vaccine, and any infections and hospitalizations documented during infancy was collected. Descriptive statistics and univariate analyses were used to assess factors associated with infection and hospitalization using SPSS (version 27.0).

**Results:**

We identified 63 infants exposed to TNF-α inhibitors in utero, 49 (77.8%) of whom received BCG vaccination within the first year of life, including 27 infants (42.8%) who were vaccinated at birth. Most infants vaccinated at birth (20/27) were exposed to TNF-α inhibitors during the third trimester. Certolizumab was the most frequently prescribed agent. Over half of the children in this cohort experienced at least three infections during infancy; however, none developed disseminated tuberculosis. Exposure to TNF-α inhibitors did not demonstrate an increased risk of infection or hospitalization during the first year of life.

**Conclusion:**

BCG vaccination at birth did not result in disseminated tuberculosis, even among infants exposed to TNF-α inhibitors during the third trimester.

## 1 Introduction

Patients with autoimmune rheumatic diseases are at risk of various pregnancy-related complications. Therefore, controlling disease activity is essential to prevent maternal and fetal morbidity (1–3). Tumor necrosis factor-alpha (TNF-α) inhibitors are effective and generally considered safe during pregnancy for treating rheumatoid arthritis (RA), spondyloarthropathy (SpA), and inflammatory bowel disease (IBD) (4–10). Nevertheless, these agents can cross the placenta and may pose risks to the fetus. Among TNF-α inhibitors, infliximab and adalimumab are substantially transferred across the placenta, whereas certolizumab is transferred at much lower levels (11).

TNF inhibitors with high placental transfer can cause elevated serum levels in neonates when administered after gestational week 30 (11–13). When used beyond the third trimester, these agents may persist in the blood of infants for 6 months and, in some cases, up to 1 year after birth (11–17). Therefore, concerns about fetal immunosuppression have emerged, particularly regarding the risk of postnatal infections and adverse reactions to live vaccines. According to recent guidelines, for infants exposed during pregnancy to TNF inhibitor biologics that cross the placenta in the later stages, Bacillus Calmette–Guérin (BCG) immunization should be deferred for 6 months. This applies to infliximab, adalimumab, and golimumab when administered after 20 weeks of gestation, and to etanercept after 32 weeks. In contrast, certolizumab pegol exhibits negligible placental transfer, so the standard vaccination timeline remains appropriate (18–20).

The BCG vaccine is a live attenuated vaccine recommended by the World Health Organization for all neonates at birth in countries with a high prevalence of tuberculosis (TB) (21). A case of disseminated BCG disease has been reported in a 4-month-old infant vaccinated at 3 months of age following in utero exposure to infliximab (22). In contrast, other reports have documented no adverse events in infants vaccinated with BCG before 6 months of age despite similar exposure (23–25).

Given the conflicting data, this study aimed to assess the incidence of disseminated BCG disease and other infections following BCG vaccination in infants born to mothers who received TNF-α inhibitors during pregnancy.

## 2 Materials and methods

### 2.1 Data collection

This retrospective study used data from electronic medical records of patients who received care at the Pregnancy and Rheumatic Disease Clinic and the IBD and Pregnancy Clinic at Hamad General Hospital (HGH) between July 2016 and July 2023. HGH is the largest tertiary hospital in the State of Qatar. Medical records of pregnant women diagnosed with autoimmune diseases, including RA, SpA, and IBD, were screened. Only women (and their infants) exposed to TNF-α inhibitors during pregnancy were eligible for inclusion. Infants were included if they had reached at least 6 months of age by the time of data collection. We excluded women who were lost to follow-up during pregnancy and those who had no pregnancy outcome data.

Data collected included demographics, age at pregnancy, rheumatic disease diagnosis, comorbidities, medications used during pregnancy, obstetric and peripartum details, neonatal outcomes, age at BCG vaccination, and adverse reactions to the vaccine, including local BCG site reactions, regional lymphadenitis, post-vaccination fever, and disseminated TB. In addition, data on infectious events, hospitalization, and ICU admission were collected. Upper respiratory tract infection was recorded when an infant had a clinical diagnosis of upper respiratory infection; in the absence of microbiologic confirmation, cases were not sub-classified as viral or bacterial. Fever was considered a presenting sign rather than an independent diagnosis and was classified according to the associated clinical syndrome when applicable.

Datasheets were coded with a serial number per pregnancy. No participant interviews or follow-up were required for the study.

### 2.2 Outcomes

The primary outcome was the incidence of disseminated TB following BCG vaccination among infants born to mothers who received TNF-α inhibitors during pregnancy. Secondary outcomes included neonatal outcomes, the frequency of infections during infancy, and factors associated with infection and hospitalization within the first year of life.

### 2.3 Approval

This study adhered to the principles of the Declaration of Helsinki and Good Clinical Practice and complied with the laws and regulations of the Ministry of Public Health, Qatar. The study was approved by the Medical Research Center at HGH, with institutional review board approval (MRC-01-24-209). Patient consent was waived by the review board due to the retrospective design. All data were anonymized.

### 2.4 Statistical analysis

Descriptive statistics were used to summarize demographic characteristics, BCG vaccination–related parameters, infection rates, and pregnancy outcomes. Normally distributed continuous variables are presented as mean ± standard deviation, whereas non-normally distributed variables are reported as median and interquartile range. Categorical variables are presented as frequencies and percentages. Rates are reported with 95% confidence intervals to reflect the precision of point estimates. Univariate analyses were performed to evaluate associations between maternal and perinatal factors and infection or hospitalization during the first year of life, with odds ratios (ORs) and 95% confidence intervals (CIs) calculated for each factor. Statistical analyses were conducted using SPSS version 27.0 (IBM Inc., Armonk, NY, United States).

## 3 Results

In total, 63 pregnancies in 55 women were included in the study. The mean (±SD) maternal age at pregnancy was 33 ± 4.0 years. The average (±SD) duration of TNF-α inhibitor intake during pregnancy was 7 ± 2.4 months. The mean (±SD) gestational period was 37.9 ± 2.0 weeks, and the average (±SD) birth weight of the infants was 3.0 ± 0.5 kg. During pregnancy, the most common indication for TNF-α inhibitor therapy was RA (33.3% of pregnancies), followed by SpA (20.6%), Crohn’s disease (19.0%), psoriatic arthritis (14.3%), ulcerative colitis (11.1%), and Behçet’s disease (1.6%). Concomitant medications during pregnancy included sulfasalazine (14.3%), hydroxychloroquine (12.7%), and azathioprine (20.6%) (Table 1).

**Table 1:** Maternal and infant characteristics during pregnancy.

Characteristic	Mean ± SD	Frequency (*n*, %)
Maternal age at pregnancy (years)	33 ± 4.0	—
Duration of TNF-α inhibitor intake (months)	7 ± 2.4	—
Rheumatological diseases		
Rheumatoid arthritis	—	21 (33.3%)
Spondyloarthritis	—	22 (34.9%)
Inflammatory bowel disease	—	19 (30.1%)
Behçet’s disease	—	1 (1.6%)
Background medications		
Sulfasalazine	—	9 (14.3%)
Hydroxychloroquine	—	8 (12.7%)
Azathioprine	—	13 (20.6%)
Pregnancy morbidities		
Pre-eclampsia	—	2 (3.2%)
Gestational diabetes	—	15 (23.8%)
Hypothyroidism	—	5 (7.9%)
Delivery types		
TERM	—	54 (85.7%)
PRE-TERM	—	8 (12.7%)
PROM	—	4 (6.3%)
NVD	—	26 (41.3%)
C-section	—	31 (49.2%)
Assisted	—	8 (12.7%)
Neonatal outcomes		
Newborn birth weight (kg)	3.0 ± 0.5	—
Low birth weight	—	8 (13.7%)
Congenital anomalies	—	0 (0%)
NICU admission	—	13 (20.6%)
Neonatal jaundice	—	18 (28.6%)

SD, standard deviation; TNF-α, tumor necrosis factor alpha; TERM, term delivery; PRE-TERM, preterm delivery; PROM, premature rupture of membranes; NVD, normal vaginal delivery; C-section, cesarean section; NICU, neonatal intensive care unit.

Pregnancy-related comorbidities included pre-eclampsia in 3.2% of cases, gestational diabetes mellitus in 23.8%, and hypothyroidism in 7.9%. Term deliveries occurred in 85.7% of cases, whereas 12.7% were preterm. Mode of delivery included normal vaginal delivery (41.3%), cesarean section (49.2%), and assisted deliveries (12.7%). Premature rupture of membranes was reported in 6.3% of cases. Among neonatal outcomes, low birth weight was observed in 13.7% of infants; however, no congenital anomalies were reported. Neonatal intensive care unit (NICU) admission was required in 20.6% of cases; all infants were successfully discharged, with no recorded mortality. Neonatal jaundice occurred in 28.6% of cases (Table 1). Among neonates requiring NICU admission, the most common indications were sepsis (6 cases; all culture-negative, often in the setting of maternal chorioamnionitis), respiratory distress (5 cases, including one with asphyxia and hypoxia requiring non-invasive ventilation and others requiring CPAP), and prematurity (3 infants). Less frequent reasons included single cases of transient tachypnea of the newborn, ABO incompatibility with hyperbilirubinemia requiring phototherapy, hypoglycemia, and vomiting with abdominal distension. During the first trimester, 79.4% of pregnancies were exposed to TNF-α inhibitors, with certolizumab as the most commonly used agent (39.7%). Other TNF-α inhibitors included infliximab (15.9%), adalimumab (12.7%), etanercept (9.5%), and golimumab (1.6%), whereas 20.6% of pregnancies had no TNF-α inhibitor exposure. Exposure to TNF-α inhibitors remained high during the second and third trimesters, with certolizumab consistently the predominant agent. The full distribution across trimesters is presented in Table 2.

**Table 2:** Distribution of biologic treatments across trimesters in all cases.

TN inhibitors	1st trimester [*n*, frequency (%)]	2nd trimester [*n*, frequency (%)]	3rd trimester [*n*, frequency (%)]
None	13 (20.6)	6 (9.5)	7 (11.1)
Any TNF-α inhibitor	50 (79.4)	57 (90.5)	56 (88.9)
Etanercept	6 (9.5)	5 (7.9)	0 (0)
Adalimumab	8 (12.7)	9 (14.3)	9 (14.3)
Certolizumab	25 (39.7)	33 (52.4)	36 (57.1)
Infliximab	10 (15.9)	8 (12.7)	9 (14.3)
Golimumab	1 (1.6)	2 (3.2)	2 (3.2)

TNF-α, tumor necrosis factor alpha.

A total of 63 infants were included in the study. Of these, 49 (77.8%) received the BCG vaccine, whereas 14 (22.2%) did not; only 27 infants (42.8%) were vaccinated at birth. Notably, most infants vaccinated at birth (20/27) had been exposed to TNF-α inhibitors during the third trimester, with certolizumab as the predominant therapy. The frequency of infection encounters varied across infants, with the largest group (42.8%, *n* = 27) experiencing more than three infection episodes during the first year of life. Among 170 recorded infections, the most common diagnosis was upper respiratory tract infection (91; 53.5%), followed by otitis media (25; 14.7%), gastritis (17; 10.0%), fever (9; 5.3%), urinary tract infection (8; 4.7%), bronchiolitis (7; 4.1%), oral candidiasis (6; 3.5%), COVID-19 (4; 2.4%), and skin infection (3; 1.8%) (Table 3). Fever was recorded as a presenting feature rather than an independent infectious diagnosis when applicable.

**Table 3:** Timing of BCG vaccination and frequency of infection encounters.

Characteristics	Frequency (*n*, %)
BCG vaccine administration (*n* = 63)	
Infants who received BCG	49 (77.8)
Infants vaccinated at birth	27 (42.8%)
Median age at BCG administration (months), median (IQR)	0 (0–6)
Number of infection encounters	
0	9 (14.3)
1	9 (14.3)
2	7 (11.1)
3	11 (17.5)
>3	27 (42.8)

BCG, Bacillus Calmette–Guérin; IQR, interquartile range.

Importantly, no cases of disseminated TB or other serious BCG-related complications were observed, apart from two self-limited local injection-site reactions. No comparative analysis between infants vaccinated at birth and those vaccinated later or not vaccinated was possible for disseminated tuberculosis because no primary outcome events occurred.

### 3.1 Factors associated with infection and hospitalization

Table 4 summarizes the associations between maternal and pregnancy-related factors—including third-trimester exposure to TNF-α inhibitors—and the risks of infection and hospitalization. For infection outcomes, none of the evaluated predictors showed a statistically significant association. Third-trimester TNF-α inhibitor exposure was identical among infants with and without infections (88.9% vs. 88.9%; *p* = 1.000). Third-trimester certolizumab exposure was not associated with either infection or hospitalization. Likewise, other maternal and obstetric variables—including gestational diabetes, preterm birth, cesarean delivery, premature rupture of membranes, and underlying rheumatologic disease or inflammatory bowel disease—did not differ significantly between groups.

**Table 4:** Univariate analysis of factors associated with infection and hospitalization during the first year of life.

Predictor	Hospitalized, *n* (%) (*n* = 23)	Not hospitalized, *n* (%) (*n* = 40)	*p*-value	OR (95% CI)
Exposure to TNF-α inhibitors (3rd trimester)	23 (100.0)	33 (82.5)	0.999^†^	10.507 (0.575–192.106)
Exposure to certolizumab (3rd trimester)	16 (69.6)	20 (50.0)	0.131	2.286 (0.774–6.751)
Gestational diabetes	7 (30.4)	8 (20.0)	0.352	1.750 (0.538–5.687)
Preterm birth	6 (26.1)	2 (5.1)	0.031	6.529 (1.193–35.750)
Cesarean section	15 (65.2)	17 (43.6)	0.103	0.412 (0.142–1.197)
Premature rupture of membranes	3 (13.0)	1 (2.6)	0.143	5.700 (0.556–58.411)
Rheumatoid arthritis	8 (34.8)	13 (32.5)	0.853	1.108 (0.375–3.273)
Spondyloarthropathy	8 (34.8)	14 (35.0)	0.986	1.010 (0.344–2.962)
Inflammatory bowel disease	6 (26.1)	13 (32.5)	0.594	0.733 (0.234–2.296)

Predictor	Infection, *n* (%) (*n* = 54)	No infection, *n* (%) (*n* = 9)	*p*-value	OR (95% CI)
Exposure to TNF-α inhibitors (3rd trimester)	48 (88.9)	8 (88.9)	1.000	1.000 (0.106–9.444)
Exposure to certolizumab (3rd trimester)	31 (57.4)	5 (55.6)	0.917	1.078 (0.260–4.466)
Gestational diabetes	13 (24.1)	2 (22.2)	0.904	1.110 (0.205–6.019)
Preterm birth	7 (13.2)	1 (11.1)	0.862	1.217 (0.131–11.273)
Cesarean section	28 (52.8)	4 (44.4)	0.643	1.400 (0.338–5.798)
Premature rupture of membranes	4 (7.5)	0 (0.0)	0.525^†^	1.727(0.170–17.300)
Rheumatoid arthritis	19 (35.2)	2 (22.2)	0.451	1.900 (0.358–10.071)
Spondyloarthropathy	18 (33.3)	4 (44.4)	0.520	0.625 (0.149–2.615)
Inflammatory bowel disease	16 (29.6)	3 (33.3)	0.823	0.842 (0.187–3.789)

The † symbol indicates odd ratios and 95% CI were estimated using continuity correction because one cell contained zero.

In contrast, for hospitalization outcomes, preterm birth was significantly associated with higher odds of hospitalization (26.1% vs. 5.1%; OR 6.53, 95% CI 1.19–35.75; *p* = 0.031). No other maternal or pregnancy-related factors—including third-trimester TNF-α inhibitor exposure, gestational diabetes, cesarean delivery, premature rupture of membranes, or underlying rheumatologic disease or inflammatory bowel disease—were significantly associated with hospitalization.

## 4 Discussion

Given the increasing use of TNF-α inhibitors for managing autoimmune diseases during pregnancy, understanding the safety of vaccinations in this context is of paramount importance. This study is one of the few to assess the safety of administering the BCG vaccine to infants whose mothers underwent TNF-α inhibitor therapy for autoimmune diseases during pregnancy. Of 63 pregnancies, 49 infants (77.8%) received the BCG vaccine within their first year, including 27 (42.8%) who were vaccinated at birth. Among those vaccinated at birth, most (*n* = 20) had been exposed to TNF-α inhibitors during the third trimester, predominantly certolizumab. Although over half of the children in this cohort experienced at least three infections during infancy, no cases of disseminated TB were observed. Similarly, no serious adverse events occurred in infants who received the BCG vaccine at birth or later during infancy. These findings are reassuring, but they should be interpreted cautiously because of the retrospective design, the limited sample size, and the potential influence of obstetric and perinatal factors on neonatal outcomes.

Immunoglobulin G (IgG) is delivered across the chorionic villi by the neonatal Fc receptor, providing passive immunity to the infant. This placental transfer increases progressively from the second trimester until delivery, leading to substantial fetal exposure during the third trimester (26). TNF-α inhibitors belong to the IgG1 subclass. Julsgaard et al. (17) showed that levels of infliximab and adalimumab are higher in fetal cord blood at delivery than in maternal blood, leading to the recommendation of delaying live vaccines for up to 1 year unless drug clearance is identified. Consequently, most guidelines suggest delaying live attenuated vaccines in infants with in utero exposure to TNF-α inhibitors for 6–12 months (18–20). Certolizumab shows the lowest level of placental transfer, suggesting negligible in utero fetal exposure during the third trimester and supporting its continued safe use throughout pregnancy when indicated (27).

Although studies on the safety of live attenuated vaccine administration generally do not report serious adverse events, isolated cases of fatal disseminated TB infections after BCG vaccination in infants exposed to TNF-α inhibitors in utero have been documented (28, 29). For instance, one case report described a fatal disseminated TB following BCG vaccination in a 3-month-old infant whose mother, affected by Crohn’s disease, had been treated with infliximab during pregnancy (28). In addition, a freedom of information request to the United Kingdom Medicines and Healthcare Products Regulatory Agency identified four additional cases of life-threatening disseminated BCG infections in infants with in utero exposure to TNF-α inhibitors, including two cases related to infliximab, one to adalimumab, and one to an unspecified TNF-α inhibitor (29).

Conversely, our findings align with a few recent retrospective studies that reported no adverse events despite infants born to mothers exposed to TNF-α inhibitors during pregnancy receiving BCG vaccination. This remained consistent even when vaccination was administered before 6 months of age (24, 28–30). One such study, using data from the French Health Insurance Database, revealed that among infants with in utero exposure to anti-TNF therapy, 64 of 88 (73%) received the BCG vaccine before 6 months of age. Importantly, no severe BCG-related adverse events were observed within the first year of life. In that cohort, maternal anti-TNF therapy included infliximab in 39 pregnancies and adalimumab in 47, with two additional cases involving sequential treatment with infliximab followed by adalimumab (24). Another brief report described nine of 90 infants with in utero exposure to TNF-α inhibitors who received the BCG vaccine within the first month of life; only one infant developed mild lymphadenopathy, which resolved spontaneously without intervention. In that case, the mother had been treated with infliximab (31). The relatively high number of clinically suspected neonatal sepsis cases should be interpreted in the context of coexisting maternal and neonatal risk factors. In our cohort, these events were not microbiologically confirmed, and affected infants had short neonatal intensive care unit stays with complete recovery. Maternal chorioamnionitis in the corresponding cases was also diagnosed clinically, with negative culture results. More broadly, outcomes such as infection and hospitalization may have been influenced by prematurity, low birth weight, premature rupture of membranes, maternal diabetes, and mode of delivery rather than by TNF-α inhibitor exposure alone. In our cohort, preterm birth was the only significant predictor of hospitalization, whereas no maternal or pregnancy-related factors were associated with infection or hospitalization. Given the small sample size and retrospective design, this finding should be interpreted with caution and is not generalizable.

Given the mixed findings across studies, larger prospective cohort studies are needed to evaluate the long-term safety of BCG vaccination in this population, particularly in endemic areas.

Nevertheless, vaccination strategies for TNF-α inhibitor-exposed children are often not implemented in accordance with current guidelines (18–20, 32–35). For example, Luu et al. (24) reported that 73% of vaccinations did not comply with recommendations to delay live attenuated vaccines until 6 months in TNF-α inhibitor-exposed children. In our study, we found that many infants who received BCG at birth were exposed to TNF-α inhibitors during the third trimester. In addition, there was no clear documentation regarding the reasons for the missing BCG vaccination in 14 infants. This non-adherence to live vaccination guidelines may reflect practices among pediatricians and obstetricians rather than rheumatologists and IBD specialists, likely due to misunderstandings regarding in utero exposure.

This study had some limitations worth noting. First, the sample size may not be sufficient to generalize the findings to all infants with in utero exposure to TNF-α inhibitors who received BCG at birth. Second, potential biases may have arisen in the data collection process, such as incomplete medical records. Third, due to the frequent use of corticosteroids across inflammatory autoimmune diseases, accurately capturing steroid exposure from medical records was challenging. However, in non-IBD pregnancies, local practice guidelines mandate avoiding corticosteroid use during pregnancy. Finally, the long-term effects of BCG vaccine administration at birth beyond infancy were not assessed, highlighting the need for further studies to fully understand its impact in this population.

In this retrospective cohort, neonatal BCG vaccination in infants with in utero exposure to TNF-α inhibitors was not associated with observed cases of disseminated tuberculosis. Secondary infectious outcomes were not clearly increased in relation to third-trimester certolizumab exposure; however, these findings should be interpreted cautiously given the small sample size, potential confounding by perinatal factors, and limited follow-up. Multicenter prospective studies are needed before firm conclusions can be drawn regarding the optimal timing of BCG vaccination in this population.

## Data Availability

The raw data supporting the conclusions of this article will be made available by the authors, without undue reservation.
